# Prevalence of metabolic syndrome (MetS) in Chinese subjects gradually increased with impaired glucose homeostasis: a multicenter, clinical based, cross-sectional study

**DOI:** 10.1186/1471-2458-12-675

**Published:** 2012-08-20

**Authors:** Yufei Xiang, Gan Huang, Weidong Zhou, Zhihong Che, Pengcheng Zhou, Zhiguang Zhou

**Affiliations:** 1 Diabetes Center, 2nd Xiangya Hospital, and Institute of Metabolism and Endocrinology, Key Laboratory of Diabetes Immunology, Ministry of Education, Central South University, Changsha, Hunan, 410011, China

**Keywords:** Metabolic syndrome, Diabetes, Impaired fasting glucose, Impaired glucose tolerance

## Abstract

**Background:**

Metabolic Syndrome (MetS) is a high risk factor for Cardiovascular Diseases (CVD). We estimated to investigate how MetS prevalence by glucose homeostasis varies across different age and gender groups.

**Methods:**

We studied 9257 Chinese subjects over the age of 15 years in two cross-sectional surveys in 2006. With oral glucose tolerance test (OGTT) test, 2341 subjects were normal glucose tolerance (NGT), and 5448 were diagnosed as having type 2 diabetes (T2D). All other 1468 subjects were considered to be impaired fasting glucose (IFG) or impaired glucose tolerance (IGT) subjects. Diabetes was diagnosis by WHO99 criteria. We used modified NCEP-III criteria for the diagnosis of MetS.

**Results:**

The prevalences of MetS in the male NGT, IFG/IGT and T2D groups were 25.9% (404/1559), 65.6% (769/1172), and 73.5% (2483/3376), respectively. The prevalences of MetS in the female NGT, IFG/IGT and T2D groups were 13.4% (105/782), 51.0% (151/296), and 75.4% (1563/2072), respectively. The prevalence of MetS in the male IFG/IGT group gradually decreased from 73.26% to 41.08% in subjects over the age of 30 years. The prevalence of MetS in the female IFG/IGT group gradually increased from 30% to 75% with aging.

**Conclusions:**

The prevalence of MetS in subjects with different glucose tolerances in China was high and gradually increased with impaired glucose homeostasis both in males and females.

## Background

The current understanding of Metabolic Syndrome (MetS) is based on a cluster of conditions: dysglycemia, dyslipidemia, hypertension, and the procoagulant state [1,2]. Accumulated data suggest that MetS is an immediate precursor to cardiovascular disease (CVD) [3]. Moreover, subjects with diabetes are known to be at greater risk for CVD than subjects without diabetes [4]. The prevalence of MetS in the world is rapidly increasing [4]. According to NCEP-ATPIII criteria [5], it is approximately 21.0% in Oman [6], 23.0% in Arab Americans [7], 38.8% in men and 22.2% in women in Finland [8], 34.5% [9] in adults but 5.2% in youth [10] in the US and from 9.8% to 46.5% in developing countries [11].

With the economic development of China, the prevalence of MetS has increased significantly. In 1992, according to a national investigation, the prevalence of MetS in mainland China was about 14.4% in men and 20% in women according to NCEP-ATPIII criteria [12]. In 2000–2001, another national survey showed that approximately 13.7% of adults aged 35–74 years had MetS as defined by NCEP-ATP III criteria [13]. In 2008, the prevalence of MetS in China increased to 23.3% according to the modified NCEP-ATPIII criteria [14]. MetS has become an important public health problem in China.

According to the latest national study, diabetes is prevalent in China. The prevalences of MetS in T2D subjects and IFG/IGT subjects now are worth to study for the prediction of CVD in those high risk populations [15]. To date, a study regarding MetS having a large sample size with subjects of different glucose homeostasis status has never been undertaken in China. Therefore, we designed this study to investigate the prevalence of MetS and its components in subjects of different glucose homeostasis status with an unusual big sample size (n = 9257), including those subjects with Normal Glucose Tolerance (NGT), Impaired Fasting Glucose (IFG)/Impaired Glucose Tolerance (IGT) and Type 2 Diabetes (T2D).

## Methods

### Research design

This was a cross-sectional study and included subjects from two large-sample investigations: the Railway System Staff Investigation and the LADA China multicenter investigation. The Railway Staff investigation was carried out in November and December of 2006. Subjects who were 18–75 years old from the Railway System were recruited for a diabetes screening. The inclusion criteria for this study including: 1) age 18–75 years old male and female; 2) willing to accept Oral Glucose Tolerance Test (OGTT). The exclusion criteria for this study including: 1) malignancy; 2) pregnancy. The LADA China multicenter investigation was initiated in June of 2006 and finished in January of 2007 in 46 hospitals around China. The inclusion criteria for this study including: 1) diagnosis of diabetes (WHO 99 criteria) at age ≥ 15.0 years, 2) disease duration of less than 1 year, 3) no ketoacidosis in the first 6 months after the diagnosis of diabetes and 4) insulin independence (usage of insulin less than 1 month) for 6 months after diagnosis. Exclusion criteria were 1) secondary diabetes, 2) diabetes in pregnancy and gestational diabetes, and 3) malignancy. Subjects greater than 15 years old and those newly diagnosed with T2D were further screened for islet autoantibodies. Fasting blood samples were drawn and screened for glucose. Overall, 2341 subjects whose fasting plasma glucose (FPG) was lower than 5.6 mmol/L were placed into the normal glucose tolerance (NGT) group. Subjects with an FPG between 5.6 mmol/L and 7.0 mmol/L were given an oral glucose tolerance test (OGTT). In total, 5448 subjects with an FPG greater than 7.0 mmol/L or postprandial plasma glucose (PPG) greater than 11.1 mmol/L were diagnosed with T2D. All other subjects (1468 subjects) were considered as impaired fasting glucose (IFG) or impaired glucose tolerance (IGT) subjects. MetS was diagnosed according to the modified National Cholesterol Education Program Third Adult Treatment Panel (NCEP-ATPIII) criteria [5].

The Ethics Review Committee/Institutional Review Board of the Second Xiangya Hospital of Central South University approved the study protocol. The study was conducted in accordance with the Declaration of Helsinki. All participants provided written informed consent.

### Sample collection and diagnosis criteria

After collecting a detailed history, including duration of diabetes, past medical history, family history, and medication history, fasting blood samples were collected from each subject.

Basic characteristics were collected by well trained clinical physician in each center, including age, height (Ht), weight (Wt), body mass index (BMI), waist circumference (WC), hip circumference (HC), waist to hip ratio (WHR), systolic blood pressure (SBP), and diastolic blood pressure (DBP). We required all venous blood samples to be tested immediately for fasting glucose level and lipids. Triglycerides (TG), total cholesterol (TC), high-density lipid cholesterol (HDL-Ch), low-density lipid cholesterol (LDL-Ch) and fasting plasma glucose (FPG) were recorded for each patient.

Obesity (BMI ≥ 30 kg/m2) and overweight (BMI ≥ 25 kg/m2) were defined by WHO 97 criteria [16]. Hypertension was defined as SBP ≥ 140 mmHg or DBP ≥ 90 mmHg or current use of antihypertensive medications (by WHO-ISH 99 criteria) [17]. Abnormal blood pressure, central obesity, abnormal TG and abnormal HDL-ch were defined according to the modified NCEP-ATPIII criteria. MetS was diagnosed with the modified NCEP-ATPIII criteria [5].

### Statistical analysis

Statistical analysis was performed with SPSS statistical software (version 13; SPSS, Chicago, IL). Normally distributed data were expressed as the means ± SD. Variables with a skewed distribution were reported as median (range). Logarithmic transformations were applied to non-normally distributed parameters. Frequency differences were compared using the *χ*2 test or Fisher’s exact test when appropriate. Variance analysis was used for comparisons of means between groups by the independent *t* test, the t’ test or one-way ANOVA when appropriate. The nonparametric Wilcoxon test was used for parameters that were not normally distributed after transformation. Pairware comparison was used by the one-way ANOVA Post Hoc test. OR was calculated by complex samples logistic regression methods. P values less than 0.05 were considered statistically significant.

## Results

### Basic characteristics of subjects

In total, there were 6107 male subjects, including 1559 NGT, 1172 IFG/IGT, and 3376 T2D subjects, and 3150 female subjects, including 782NGT, 296 IFG/IGT and 2072 T2D subjects. We compared basic clinical characteristics among the subjects (Table 1). In males, BMI, SBP and HDL-Ch were similar between the IFG/IGT and T2D subjects. All other indexes increased or decreased gradually from the NGT to the IFG/IGT to the T2D groups (P<0.001 for all comparisons). In females, Ht was similar among the NGT, IFG/IGT and T2D groups. Wt, HC and HDL-Ch were similar between the IFG/IGT and T2D groups but lower in the NGT group. Other clinical characteristics increased or decreased gradually from the NGT to the IFG/IGT to the T2D groups (P<0.05 for all comparisons).

**Table 1:** Clinical characteristics of the NGT, IFG/IGT and T2D subjects

	**Male**				**Female**			
	**NGT**	**IFG/IGT**	**T2D**	**P value**	**NGT**	**IFG/IGT**	**T2D**	**P value**
	**(N = 1559)**	**(N = 1172)**	**(N = 3376)**		**(N = 782)**	**(N = 296)**	**(N = 2072)**	
Age (yrs)	47.09 ± 8.47	49.89 ± 6.66^***^	52.54 ± 12.07^***###^	<0.001	43.18 ± 8.65	46.80 ± 6.64^***^	48.90 ± 12.07^***###^	<0.001
Ht (cm)	168.22 ± 7.19	168.22 ± 7.59	169.81 ± 6.89^***###^	<0.001	158.04 ± 7.75	158.53 ± 5.49	157.80 ± 5.54	0.133
Wt (kg)	67.32 ± 9.76	71.30 ± 9.53^***^	72.33 ± 11.50^***##^	<0.001	56.33 ± 8.19	59.71 ± 8.86^***^	60.81 ± 9.97^***^	<0.001
BMI (kg/m^2^)	23.74 ± 2.92	25.12 ± 2.86^***^	25.01 ± 3.35^***^	<0.001	22.50 ± 3.08	23.74 ± 3.19^***^	24.39 ± 3.59^***##^	<0.001
WC (cm)	83.47 ± 8.70	87.56 ± 8.23^***^	89.87 ± 9.81^***###^	<0.001	75.48 ± 7.67	78.92 ± 8.03^***^	84.47 ± 9.70^***###^	<0.001
HC (cm)	95.61 ± 6.30	97.72 ± 6.48^***^	97.25 ± 8.56^***^	<0.001	93.06 ± 5.92	95.15 ± 6.13^***^	95.17 ± 8.91^***^	<0.001
WHR	0.8717 ± 0.05382	0.8943 ± 0.05194^***^	0.9251 ± 0.06421^***###^	<0.001	0.8103 ± 0.05402	0.8287 ± 0.05390^***^	0.8877 ± 0.07221^***###^	<0.001
SBP (mmHg)	119.21 ± 17.52	127.18 ± 18.46^***^	126.55 ± 16.99^***^	<0.001	113.37 ± 17.27	121.29 ± 21.35^***^	128.64 ± 19.12^***###^	<0.001
DBP (mmHg)	78.78 ± 11.43	83.35 ± 11.10^***^	81.06 ± 10.45^***###^	<0.001	73.42 ± 11.60	77.72 ± 11.88^***^	79.56 ± 10.51^***##^	<0.001
TG (mmol/L)	1.50(0.30-28.86)	1.78(0.42-28.26)^**^	1.87(.06-34.15)^***###^	<0.001	1.06(0.26-29.31)	1.32(0.36-13.14)^**^	1.65(0.24-22.69)^***##^	<0.001
TC (mmol/L)	5.12(2.37–11.45)	5.34(2.64–14.42)^***^	4.93(.04–42.20)^***###^	<0.001	5.05(2.19–10.37)	5.22(3.40–7.74)^**^	4.93(1.03-15.85)^###^	0.002
HDL-ch (mmol/L)	1.25(0.36–3.82)	1.21(0.41–2.65)^*^	1.10(0.09–7.58)^**^	0.007	1.42(0.65–2.52)	1.35(0.54–2.23)^*^	1.26(0.04-11.11)^*^	0.019
LDL-ch (mmol/L)	3.16(0.94–8.39)	3.32(1.16–10.82)^***^	2.82(0.12–12.67)^***###^	<0.001	3.12(0.53–7.50)	3.30(1.79–5.35)^**^	2.78(0.40-13.50)^***###^	<0.001
FBS (mmol/L)	5.23(3.84–5.59)	5.90(4.57–6.99)^***^	7.80(3.84–22.81)^***###^	<0.001	5.16(3.90–5.59)	5.82(4.76–6.90)^***^	8.43(2.68-16.00)^***###^	<0.001

Compared with NGT ^*^P<0.05, ^**^P<0.01, ^***^P<0.001;

Compare with IFG/IGT ^#^P<0.05, ^##^P<0.01, ^###^P<0.001.

### The prevalences of MetS in different gender subgroups

The overall prevalences of MetS in the NGT, IFG/IGT and T2D groups were 21.7% (20.1–23.4), 62.7% (60.2–65.2), and 74.3% (73.1–75.4), respectively. Considering gender differences, as shown in Table 2, The prevalences of MetS in the male NGT, IFG/IGT and T2D groups were 25.9% (23.7–28.1), 65.6% (62.9–68.3), and 73.5% (72.1–75.0), respectively. The prevalences of MetS in the female NGT, IFG/IGT and T2D groups were 13.4% (11.03–15.82), 51.0% (45.3–56.8), and 75.4% (73.6–77.3), respectively.

**Table 2:** Prevalence of the MetS in different gender group

	**Overall**			**Male**			**Female**		
	**NGT**	**IFG/IGT**	**T2D**	**N T**	**IFG/IGT**	**T2D**	**NGT**	**IFG/IGT**	**T2D**
N	2341	1468	5448	1559	1172	3376	782	296	2072
MetS	509	920	4046	404	769	2483	105	151	1563
95%CI	21.7%	62.7%	74.3%	25.9%	65.6%^***^	73.6%^***###^	13.4%^&&&^	51.0%^***&&&^	75.4%^***###^
	(20.1–23.4)	(60.2–65.2)	(73.1–75.4)	(23.7–28.1)	(62.9–68.3)	(72.1–75.0)	(11.03–15.82	(45.3-56.8)	(73.6-77.3)
OR	Ref	6.078	10.435	Ref	5.486	7.956	Ref	6.752	20.018
		(5.261–7.020)	(9.297–11.711)		(4.650–6.472)	(6.940–9.120)		(4.971-9.172)	(15.928-25.157)

Compared with NGT ^*^P<0.05, ^**^P<0.01, ^***^P<0.001;

Compared with IFG/IGT ^#^P<0.05, ^##^P<0.01, ^###^P<0.001;

Compared with male group ^&^P<0.05, ^&&^P<0.01, ^&&&^P<0.001.

### The prevalences of MetS in different age subgroups

We stratified the population by age (Table 3). For the male NGT group, the <20-year-old subjects had the lowest prevalence of MetS compared with the other age groups.

**Table 3:** Age-related prevalence of the MetS in subjects with different gender

	**Male**				**Female**			
	**NGT**	**IFG/IGT**	**T2D**	**P value for trends**	**NGT**	**IFG/IGT**	**T2D**	**P value for trends**
N	1559	1172	3376		782	296	2072	
<30	18.1%(15/83)	35.7%(5/14)	63.9%(115/180)^***#^	P<0.001	3.3%(3/91)^##^	30.0%(3/10)^*^	54.9%(39/71)^***^	P < 0.001
%95CI	(9.6–26.5)	(7.0–64.4)	(56.8–71.0)		(−0.4–7.0)	(−4.6–64.6)	(43.1-66.8)	
OR	Ref	2.519(0.738–8.591)	8.021(4.244–15.157)		Ref	12.571(2.129–74.233)	35.750(10.324-123.791)	
30-39	20.0%(42/210)	73.3%(63/86)^***^	75.1%(410/546)^***^	P<0.001	3.8%(4/106)^&&&^	34.8%(8/23)^***&&^	61.2%(137/224)^&&&^	P < 0.001
%95CI	(14.6–25.5)	(63.7–82.8)	(71.5–78.7)		(0.09–7.5)	(13.7–55.8)	(54.7-67.6)	
OR	Ref	10.957(6.103–19.669)	12.059(8.166–17.807)		Ref	13.600(3.644–50.754)	40.155(14.271-112.986)	
40-49	26.1%(130/499)	69.1%(237/343)^***^	74.2%(783/1055)^***^	P<0.001	15.7%(69/441)^&&&^	52.0%(93/179)^***&&&^	67.8%(328/484)^***###&^	P < 0.001
%95CI	(22.2–29.9)	(64.2–74.1)	(71.6–76.9)		(12.2–19.1)	(44.6–59.4)	(63.6-72.0)	
OR	Ref	6.346(4.683–8.601)	8.171(6.409–10.417)		Ref	5.830(3.948–8.610)	11.336(8.232-15.609)	
50-59	28.0%(210/750)	64.2%(457/712)^***^	74.1%(754/1018)^***###^	P<0.001	19.7%(28/142)^&^	55.0%(44/80)^***^	79.9%(563/705)^***###&&^	P < 0.001
%95CI	(24.8–31.2)	(60.7–67.8)	(71.4–76.8)		(13.1-26.3)	(43.9-66.1)	(76.9-82.8)	
OR	Ref	4.608(3.694-5.749)	7.344(5.940-9.081)		Ref	4.976(2.720-9.104)	16.142(10.267-25.380)	
≥60	41.2% (7/17)	41.2%(7/17)	73.0%(421/577)^**##^	P<0.001	50.0%(1/2)	75.0%(3/4)	84.4%(496/588)^&&&^	P = 0.364
%95CI	(15.1–67.3)	(15.1–67.3)	(69.3–76.6)		(−5.9–6.9)	(−4.6–154.6)	(81.4-87.3)	
OR	Ref	1.000(0.255-3.919)	3.855(1.442-10.305)		Ref	3.000(0.084-107.447)	5.391(0.334-86.965)	

Compared with NGT ^*^P<0.05, ^**^P<0.01, ^***^P<0.001; Compared with IFG/IGT ^#^P<0.05, ^##^P<0.01, ^###^P<0.001; Compared with male group ^&^P<0.05, ^&&^P<0.01, ^&&&^P<0.001.

In the male NGT group, the prevalence of MetS gradually increased with aging, from 20.0% (31–40 yrs old) to 41.18% (> 61 yrs old). In the male T2D group, the MetS prevalence stayed at a very high level (more than 70%) after age 30. In the IFG/IGT group, the prevalence of MetS in the male IFG/IGT group gradually decreased from 73.26% (31–40 yrs old) to 41.18% (> 61 yrs old). Notably, in the 31-to-40-year-old male IFG/IGT group, the prevalence of MetS increased greatly when compared with the less-than-20-year-old group [73.26% (63/86) vs. 35.71% (5/14), P<0.001]. The prevalence of MetS in the female NGT, IFG/IGT and T2D groups all showed increasing trends with age. Notably, the OR of MetS in 31-to-40-year-old male subjects was 10.957 (6.103–19.669) in the IFG/IGT group and 12.059 (8.166–17.807) in the T2D group when compared with the NGT group. In female 31-to-40-year-old subjects, the OR was 13.600 (3.644–50.754) in the IFG/IGT group and, surprisingly, 40.155 (14.271–112.986) in the T2D group when compared with the NGT group.

### Metabolic component disorder among the subgroups

We studied whether the presence of glucose abnormalities is associated with the number of positive MetS as well. In both male and female subgroups, it is obviously that the number of metabolic disorders was increasing with the worsen glucose level from NGT, IFG/IGT to T2D subgroups. A correlation analysis also found that both in male and female group, the presence of MetS is associated continuously with FPG levels in both male and female group (P<0.001 for both).

We compared the abnormal metabolic components of MetS among the subgroups in Table 4. In male subjects, the abnormal blood pressure (IFG/IGT vs. T2D: 57.3% vs. 55.6%, P>0.05) did not increase with worsening glucose homeostasis between the IFG/IGT and T2D subjects, although both of them were higher than the NGT group (P<0.001 for all). However, in females, the prevalences of abnormal blood pressure and hypertension were increased from the NGT group to the IFG/IGT group to the T2D group, which was in accordance with the glucose homeostasis changes (P<0.001 for all).

**Table 4:** Metabolic disorders in different gender subjects

	**Male**				**Female**			
	**NGT**	**IFG/IGT**	**T2D**	**P value for trends**	**NGT**	**IFG/IGT**	**T2D**	**P value for trends**
N	1559	1172	3376		782	296	2072	
Abnormal Blood Pressure	556 (35.6%)	672 (57.3%)^***^	1879 (55.5%)^***^	<0.001	169 (21.4%)^&&&^	112 (37.8%)^***&&&^	1165 (56.2%)^***###^	<0.001
95%CI	(33.2–38.0)	(54.5–60.2)	(53.9–57.2)		(18.5–24.2)	(32.3–43.4)	(53.8-58.1)	
Odds Ratio	Ref	2.420(2.072–2.826)	2.255(1.993–2.552)		Ref	2.198(1.646–2.937)	4.648(3.840-5.626)	
Hypertension	382 (24.5%)	506 (43.2%)^***^	1345 (39.8%)^***#^	<0.001	108 (13.8%)^&&&^	77 (26.0%)^***&&&^	915 (44.0%)^***###&&^	<0.001
95%CI	(22.3–26.6)	(40.3–46.0)	(38.1–41.4)		(11.4–16.2)	(21.0–31.0)	(41.8-46.1)	
Odds Ratio	Ref	2.334(1.983–2.748)	2.035(1.779–2.328)		Ref	2.187(1.573–3.041)	4.931(3.954-6.149)	
Central obesity	392 (25.1%)	497 (42.2%)^***^	1716 (50.8%)^***###^	<0.001	248 (31.6%)^&&^	129 (43.2%)^***^	1459 (70.4%)^***###&&&^	<0.001
95%CI	(22.9–27.2)	(39.4–45.1)	(49.1–52.5)		(28.3–34.9)	(37.6–48.9)	(68.2-72.2)	
Odds Ratio	Ref	2.186(1.858–2.572)	3.066(2.685–3.501)		Ref	1.656(1.259–2.178)	5.097(4.269-6.086)	
Overweight & Obesity	513 (32.9%)	599 (51.1%)^***^	1614 (47.8%)^***^	<0.001	149 (19.1%)^&&&^	77 (26.0%)^**&&&^	835 (40.3%)^***###&&&^	<0.001
95%CI	(31.0–35.0)	(48.0–54.0)	(46.0–49.0)		(16.0–22.0)	(21.0–31.0)	(38.0-42.0)	
Odds Ratio	Ref	2.124(1.818–2.482)	1.862(1.643–2.110)		Ref	1.490(1.087–2.041)	2.867(2.350-3.498)	
Abnormal Triglycerides	672 (43.1%)	669 (57.1%)^***^	2010 (59.5%)^***^	<0.001	144 (18.4%)^&&&^	111 (37.5%)^***&&&^	1079 (52.1%)^***###&&&^	<0.001
95%CI	(40.6–45.5)	(54.0–59.7)	(57.7–61.1)		(15.7–21.1)	(32.0–43.1)	(49.5-53.8)	
Odds Ratio	Ref	1.749(1.501–2.038)	1.934(1.713–2.184)		Ref	2.646(1.968–3.559)	4.806(3.934-5.871)	
Abnormal HDL cholesterol	718 (46.1%)	687 (58.6%)^***^	2349 (69.6%)^***###^	<0.001	286 (36.6%)^&&&^	157 (53.0%)^***^	1455 (70.2%)^***###^	<0.001
	(43.5–48.5)	(55.5–61.2)	(67.9–71.0)		(33.8–40.6)	(48–59.4)	(68.7-72.7)	
Odds Ratio	Ref	1.653(1.419–1.926)	2.662(2.353–3.012)		Ref	1.947(1.486–2.550)	4.070(3.424-4.838)	

Compared with NGT ^*^P<0.05, ^**^P<0.01, ^***^P<0.001; Compared with IFG/IGT ^#^P<0.05, ^##^P<0.01, ^###^P<0.001; Compared with male group ^&^P<0.05, ^&&^P<0.01, ^&&&^P<0.001.

WC-diagnosed central obesity gradually increased from the NGT group to the IFG/IGT group to the T2D group in both the male and female subjects. However, BMI-defined overweight and obesity in the male IFG/IGT group was similar to the male T2D group (51.1% vs. 47.8%, P>0.05), although both of them were higher than the NGT group (32.9%, P<0.001 for both). In females, the prevalence of overweight and obesity gradually increased with worsening glucose homeostasis (P<0.001 for all).

For lipid profiles, the prevalence of abnormal TG in the male IFG/IGT group was similar to that in the T2D group (57.1% vs. 59.5%, P>0.05) but higher than that in the NGT group (43.1%, P<0.001 for all). The prevalence of abnormal HDL-Ch in both males and females was increased with worsening glucose homeostasis (P<0.001 for all).

## Discussion

Our study showed that MetS was prevalent not only in NGT subjects but also in IFG/IGT and T2D subjects. In this Chinese study population, the overall prevalence of MetS in the IFG/IGT group (62.73%) was between that of the NGT group (21.68%) and the T2D group (74.29%). In the Stop-NIDDM study, the prevalence of MetS was about 61% in the IFG/IGT population [18]. A study in Finland that used WHO criteria to diagnose MetS found that in women and men, respectively, the metabolic syndrome was seen in 10% and 15% of subjects with NGT, 42% and 64% of subjects with IFG/IGT, and 78% and 84% of subjects with T2D [19]. In the Diabetes in Germany (DIG) study, the prevalence of MetS in T2D subjects was as high as 74.4% [20]. Overall, our data are similar to those from studies of Caucasian subjects. Strikingly, compared with NGT subjects, the OR of MetS in T2D subjects is very high in both men and women. The data suggest that when glucose homeostasis is damaged, other metabolic components become worse, and the prevalence of MetS increases.

Many studies have shown that in different glucose homeostasis subgroups, the prevalence of MetS can predict CVD [20-22]. In a study carried out in Finland, cardiovascular heart disease history was related to MetS in all three subgroups: NGT, IFG/IGT, and T2D. However, previous Myocardial Infarction (MI) history was found to be significant only in the IFG/IGT group, and previous stroke was found to be significant in the T2D group [19]. Due to the high prevalences of MetS in IFG/IGT and T2D Chinese subjects, CVD and other vascular diseases, such as stroke, should be paid attention to, and this would be a heavy burden for both individual families and the public medical service system in China. However, to be note, debates and doubts about the predictive ability of MetS for adverse cardio and cerebrovascular events currently exist as well. Since our data is from cross-sectional data, readers should be cautious too.

Surprisingly, we found that in the IFG/IGT group, the prevalence of MetS gradually decreased in males greater than 30 years of age. However, in the female IFG/IGT group, the opposite trend was present. This phenomenon is the same as that previously reported for the frequency of obesity in IFG/IGT subjects [13]. We believe that this may be because more and more IFG/IGT subjects would develop T2D in old age if they already had MetS during young age. Second, because the present analyses are based on cross-sectional data, the increasing prevalence of MetS in the younger age groups could be due, in some degree, to a cohort effect. The lower prevalence of MetS in IFG/IGT subjects of greater than 61 years of age might also be due to a survivor effect, for example, if people who had MetS were more likely to die before that age and were not available for study [13]. Third, we cannot exclude that the higher prevalence noted in the younger age groups might represent an overall increase in the prevalence of MetS in recent years because of lifestyle changes in China [15]. The 31-to-40-year-old age group of IFG/IGT and T2D subjects (either male or female) had the highest OR of MetS compared with the NGT group. This finding suggests that comprehensive intervention for MetS should occur as early as 30 or before 30 years of age.

Gender differences were found in our study. According to the modified NCEP-ATPIII criteria, the prevalences of MetS in the NGT and IFG/IGT groups were higher in males than in females. Our data are different from a previous report in China that showed that prevalence of MetS was higher in women than in men (17.8% vs. 9.8%) according to 2001 ATPIII criteria [12,13]. Although most investigations in Caucasians have not found gender differences [23,24], many other reports have had results similar to our own [9,25]. We believe that different diagnostic criteria affect the prevalence of MetS in different genders. The IDF criteria use central obesity as its central diagnostic criterion. Based on our data and data from other studies [13], females in China have a higher prevalence of central obesity than males. The IDF criteria and the modified NCEP-ATPIII criteria have already changed the diagnostic criteria for central obesity and HDL-Ch according to different ethnic populations. Previous reports have shown that after changing the diagnostic criteria, which were modified for ethnic differences, the prevalence of MetS increased approximately 10% in Chinese males but decreased 2% in Chinese females [13]. After considering the change in diagnostic criteria, sex hormones should be considered to explain the differences in MetS between males and females. One study has found that higher testosterone and sex hormone binding globulin (SHBG) levels in aging males are associated with higher insulin sensitivity and a reduced risk for MetS independently of insulin levels and body composition measurements [26]. Another study has found that low testosterone and SHBG levels are strongly associated not only with components of MetS but also with MetS itself independent of BMI. Furthermore, sex hormones are associated with inflammation and body iron stores. Even in the absence of late-stage consequences, such as diabetes and cardiovascular disease, subtle derangements in sex hormones are present in the metabolic syndrome and may contribute to its pathogenesis [27]. Another interesting finding is that for different metabolic disorders, or MetS component in male subjects, quite often, including abnormal blood pressure, overweight and abnormal TG levels had a similar frequency in both IFG/IGT and T2D groups. This suggested in male, some of the MetS components worsen as early as in IFG/IGT group.

Although until now this study is the biggest to investigate MetS in different glucose homeostasis subjects in China, we acknowledge some limitations in this study. Most of the subjects lived in urban areas, and the T2D subjects were mostly from clinics and hospitals. This study was only a cross-sectional study, which cannot predict the risk of CVD directly. To be note, due to limited group size, especially the IFG subjects in female group, we didn’t separate the IFG and IGT subjects. Thus, further longitude follow up study with a even big sample size of IFG and IGT subjects should be carried out. Some inflammatory cytokines such as hs-CRP, which has been proved closely related to MetS and CVD could be measured as well.

## Conclusions

Our large sample multicenter cross-sectional investigation showed that the prevalence of MetS in Chinese was increased from NGT, IFG/IGT to T2D subjects. Early from 30 years old, intervention for MetS should be carried out to prevent further cardiovascular complications.

## Appendix A

A.1. LADA china study investigators Linong Ji, Peking University People’s Hospital (Beijing); Xiaohui Guo, Peking University First Hospital (Beijing); Tianpei Hong, Peking University Third Hospital (Beijing); Jumin Lu, The General Hospital of the People’s Liberation Army (Beijing); Zhangrong Xu, The 306th Hospital of the People's Liberation Army (Beijing); Yingsheng Zhou, Beijing Hospital (Beijing); Weiping Jia, Shanghai Jiao Tong University Affiliated 6th People’s Hospital (Shanghai); Ning Guang, Shanghai Jiao Tong University Affiliated Rui-Jin Hospital (Shanghai); Renming Hu, Hua Shan Hospital, Fudan University (Shanghai); Xin Gao, Zhongshan Hospital, Fudan University (Shanghai); Yanbing Li, The First Affiliated Hospital, Sun Yat-sen University (Guangzhou); Huazhang Yang, Guangdong General Hospital (Guangzhou); Shaoda Lin, the First Affiliated Hospital,Shantou University (Shantou); Shenren Chen, the First Affiliated Hospital, Shantou University (Shantou); Lulu Chen, Union Hospital, Tongji Medical College, Huazhong University of Science and Technology (Wuhan); Yancheng Xu, Zhongnan Hospital of Wuhan University (Wuhan); Hong Li, First Affiliated Hospital of Medical School of Zhejiang University (Hangzhou); Wei Gu, Second Affiliated Hospital of Medical School of Zhejiang University (Hangzhou); Dawang Wang, The First Affiliated Hospital of Wenzhou Medical School (Wenzhou); Qifu Li, The First Affiliated Hospital, Chongqing Medical University (Chongqin); Gangyi Yang, The Second Affiliated Hospital, Chongqing Medical University (Chongqin); Haoming Tian, West China Hospital, Sichuan University (Chengdu); Dalong Zhu, Nanjing Drum Tower Hospital, the Affiliated Hospital of Nanjing University Medical School (Nanjing); Chao Liu, Jiangsu Province Hospital, the First Affiliated Hospital with Nanjing Medical University (Nanjing); Qiuhe Ji, Xijing Hospital, Fourth Military Medical University (Xi’an); Zhongyan Shan, The First Hospital of China Medical University (Shenyang); Jianling Du, First Affiliated Hospital of Dalian Medical University (Dalian); Benli Su, Second Affiliated Hospital of Dalian Medical University (Dalian); Yan Liu, The Norman Bethune 1st hospital of Jilin University (Changchun); Huanqi Ge, The Norman Bethune 2nd hospital of Jilin University (Changchun); Yadong Sun, People’s Hospital of Jilin Province (Changchun); Qiang Li, The 2^nd^ Affiliated Hospital of Harbin Medical University (Harbin); Jiajun Zhao, Shandong Provincial Hospital (Jinan); Lingli Ouyang, The First Affiliated Hospital of Guangxi Medical University (Nanning); Yuexin Bai, The First Affiliated Hospital of Zhengzhou University (Zhenzhou); Yuanming Xue, The First People’s Hospital of Yunnan Province (Kunming); Xulei Tang, The First Affiliated Hospital of Lanzhou University (Lanzhou); Lixin Shi, The Affiliated Hospital of Guiyang Medical University (Guiyang); Xiaoyang Lai, The Second Affiliated Hospital of Nanchang University (Nanchang); Jie Liu, Shanxi Provincial People’s Hospital (Taiyuan); Liyong Yang, The First Affiliated Hospital of Fujian Medical University (Fuzhou); Huiju Zhong, Xiangya Hospital of Central South University (Changsha); Zhiguang Zhou, The Second Xiangya Hospital of Central South University (Changsha); Jianying Liu, The First Affiliated Hospital of Nanchang University (Nanchang); Jing Yang, The First Hospital of Shanxi Medical University (Taiyuan); Yongde Peng, Shanghai First People’s Hospital (Shanghai).

## Abbreviations

MetS: Metabolic Syndrome; NGT: Normal Glucose Tolerance; IFG: Impaired Fasting Glucose; IGT: Impaired Glucose Tolerance; CVD: Cardiovascular Disease; T2D: Type 2 Diabetes; NCEP-ATPIII: Third Report of the National Cholesterol Education Program (NCEP) Expert Panel on Detection, Evaluation, and Treatment of High Blood Cholesterol in Adults (Adult Treatment Panel III); LADA: Latent Autoimmune Diabetes in Adults; FPG: Fasting Plasma Glucose; Ht: Height; Wt: Weight; BMI: Body Mass Index; WC: Waist Circumference; HC: Hip Circumference; WHR: Waist to Hip Ratio; SBP: Systolic Blood Pressure; DBP: Diastolic Blood Pressure; TG: Triglycerides; TC: Total Cholesterol; HDL-Ch: High-Density Lipid cholesterol; LDL-Ch: Low-Density Lipid cholesterol.

## Competing interests

The authors declare that they have no competing interests.

## Authors’ contributions

YX researched data, wrote manuscript and contributed to discussion, GH, ZC, WZ, and PZ researched data, and ZZ designed the study, contributed to discussion, reviewed/edited manuscript. All authors read and approved the final manuscript.

## Authors’ information

Z. Zhou, MD, PhD. Professor of Endocrinology and Metabolism, is the director of the Diabetes Center, Central South University, Chief of Institute of Metabolism and Endocrinology, the Second Xiangya Hospital, and director of the Key Laboratory of Diabetes Immunology, Ministry of Education (Central South University). He is also the Associated Chairman of the Chinese Diabetes Society.

## Pre-publication history

The pre-publication history for this paper can be accessed here:

http://www.biomedcentral.com/1471-2458/12/675/prepub
